# 

**Published:** 1907-03-30

**Authors:** 


**HOW TO BECOME A CERTIFIED MIDWIFE.**
By E. L. C. Appel, M.B., B.S., B.Sc.Lond.

**THE NURSING OF SICK CHILDREN.**
By Dr. Burnet.

**SIMPLE INTRODUCTORY LESSONS IN MIDWIFERY.**
By M. Loane.

**THE MODERN NURSING OF CONSUMPTION.**
By Dr. Jane H. Walker.

**THE EXAMINATION OF THE URINE.**
By J. K. Watson, M.D., M.B., C.M., Author of "A Handbook for Nurses."

**SIMPLE SANITATION:**
**The Practical Application of the Laws of Health to Small Dwellings.**
By M. Loane. With an Introductory Chapter on Sanitary Legislation by Dr. A. Mearns Fraser, Medical Officer of Health, Portsmouth.

**PRACTICAL HINTS ON DISTRICT NURSING.**
By Miss Amy Hughes.

**FEVERS AND INFECTIOUS DISEASES.**
**Their Nursing and Practical Management.**
By William Harding, M.D.Ed., M.R.C.P.Lond., Author of "Mental Nursing," &c.

**NOTES ON PHARMACY AND DISPENSING FOR NURSES.**
New and Revised Edition.
By C. J. S. Thompson.

**SICK NURSING AT HOME.**

By Miss L. G. Moberley.

**SYLLABUS OF LECTURES TO NURSES.**

By Andrew Davidson, M.D.

### 2/6 By Post, 2/9 — NURSING MANUALS

**ELEMENTARY ANATOMY AND SURGERY FOR NURSES.**
By William McAdam Eccles, M.S.Lond., M.B., F.R.C.S., L.R.C.P. Profusely Illustrated.

**NURSING IN DISEASES OF THE THROAT, NOSE, & EAR.**
By P. Macleod Yearsley, F.R.C.S.Eng., M.R.C.S., L.R.C.P.Lond. Illustrated.

**LECTURES UPON THE NURSING OF INFECTIOUS DISEASES.**
By F. J. Woollacott, M.A., M.D.

**MEDICAL ELECTRICITY AND LIGHT TREATMENT.**
**A Practical Handbook for Nurses.**
By Kate Neale, Sister in Charge of the Actino-Therapeutic Department, Guy's Hospital. Profusely Illustrated.

**ELEMENTARY TREATISE ON THE LIGHT TREATMENT FOR NURSES.**
By James H. Sequeira, M.D.Lond. Illustrated.

**HOSPITAL SISTERS AND THEIR DUTIES.**
By Eva C. E. Lückes, Matron, London Hospital. Third Edition, enlarged and partly re-written.

**TENDENCIES TO CONSUMPTION:**
**How to Counteract Them.**
By J. D. E. Mortimer, M.B.Lond., M.R.C.S., L.S.A., Author of "Home Nursing of Sick Children."

**SPINAL CURVATURE AND AWKWARD DEPORTMENT:**
**Their Causes and Prevention in Children.**
By Dr. George Müller, Professor of Medicine and Orthopædics, Berlin. English Edition, edited and adapted by Richard Greene, F.R.C.P.Ed. With Plates and Illustrations.

### THE SCIENTIFIC PRESS, Ltd.,
**28 & 29 SOUTHAMPTON STREET, STRAND, LONDON, W.C.**
Publishers of Nursing Text-Books, Charts, and Case-Books of all Descriptions.
*CATALOGUE OF NURSING MANUALS SENT POST FREE ON APPLICATION.*

